# “It Just Kind of Feels Like a Different World Now:” Stress and Resilience for Adolescents With Type 1 Diabetes in the Era of COVID-19

**DOI:** 10.3389/fcdhc.2022.835739

**Published:** 2022-02-21

**Authors:** Maeve B. O’Donnell, Marisa E. Hilliard, Viena T. Cao, Miranda C. Bradford, Krysta S. Barton, Samantha Hurtado, Brenda Duran, Samantha Garcia Perez, Kiswa S. Rahman, Samantha Scott, Faisal S. Malik, Daniel J. DeSalvo, Catherine Pihoker, Chuan Zhou, Abby R. Rosenberg, Joyce P. Yi-Frazier

**Affiliations:** ^1^ Palliative Care and Resilience Lab, Center for Clinical and Translational Research, Seattle Children’s Research Institute, Seattle, WA, United States; ^2^ Department of Pediatrics, Division of Diabetes/Endocrinology, University of Washington School of Medicine, Seattle, WA, United States; ^3^ Cambia Palliative Care Center of Excellence, University of Washington School of Medicine, Seattle, WA, United States; ^4^ Department of Pediatrics, Texas Children’s Hospital and Baylor College of Medicine, Houston, TX, United States; ^5^ Core for Biostatistics, Epidemiology and Analytics for Research (BEAR) Core, Seattle Children’s Research Institute, Seattle, WA, United States; ^6^ Department of Psychology, University of Denver, Denver, CO, United States; ^7^ Center for Child Health, Behavior, and Development, Seattle Children’s Research Institute, Seattle, WA, United States; ^8^ Department of Pediatrics, Division of General Pediatrics, School of Medicine, University of Washington, Seattle, WA, United States

**Keywords:** type 1 diabetes, COVID-19, stress, resilience, diabetes management

## Abstract

**Purpose:**

The COVID-19 pandemic has been a major stressor for adolescents. Given the unique implications of the pandemic for youth with type 1 diabetes (T1D), who already navigate multiple stressors as a function of their chronic condition, we aimed to describe the impact of the pandemic on adolescents with T1D and describe their coping strategies and resilience resources.

**Research Method:**

In a 2-site (Seattle WA, Houston TX) clinical trial of a psychosocial intervention targeting stress/resilience, adolescents 13-18 years old with T1D ≥ 1 year and elevated diabetes distress were enrolled August 2020 – June 2021. Participants completed a baseline survey about the pandemic, including open-ended questions about the effects of the pandemic, what was helping them navigate, and how it impacted T1D management. Hemoglobin A1c (A1c) was extracted from clinical records. Free text responses were analyzed using an inductive content approach. Survey responses and A1c were summarized using descriptive statistics and associations were assessed by Chi-squared tests.

**Results:**

Adolescents (n=122) were 56% female. 11% of adolescents reported diagnosis of COVID-19 and 12% had a family member/other important person die from COVID-19 complications. Adolescents described Social Relationships, Personal Health/Safety Practices, Mental Health, Family Relationships, and School to be primary areas affected by COVID-19. Helpful resources included: Learned Skills/Behaviors, Social Support/Community, and Meaning-Making/Faith. Among participants indicating that the pandemic had an impact on their T1D management (n=35), the most commonly described areas were: Food, Self-Care, Health/Safety, Diabetes Appointments, and Exercise. Compared to adolescents who reported minimal difficulty managing T1D during the pandemic (71%), those reporting moderate to extreme difficulty (29%) were more likely to have A1C ≥ 8% (80% * vs.* 43%, p<.01).

**Conclusions:**

Results underscore the pervasive impact of COVID-19 on teens with T1D across multiple major life domains. Their coping strategies aligned with stress, coping, and resilience theories and suggest resilient responses in the face of stress. Despite experiencing pandemic-related stressors in many areas, diabetes-related functioning was relatively protected for most teens, highlighting their diabetes-specific resilience. Discussing the pandemic impact on T1D management may be an important focus for clinicians, especially for adolescents with diabetes distress and above-target A1C.

## Introduction

Adolescents with type 1 diabetes (T1D) are disproportionately affected by stress. Over one-third report high stress about their diabetes (1), with serious implications for mental and physical heath. High diabetes distress is associated with higher A1c and an increased risk of developing psychological disorders (2, 3). The COVID-19 pandemic is widely recognized as a global and potentially traumatic stressor (4) and has been linked with high rates of stress, loneliness, and increased risk of depression for teens (5). Little is known, however, about the impact for teens managing a chronic health condition, such as T1D.

While there is no existing literature on the impact of the COVID-19 pandemic on the psychosocial well-being of adolescents with diabetes, research in adults with diabetes (both type 1 and type 2) suggests a significant impact on psychosocial health and diabetes management. Due, likely in part, to the increased risk of morbidity and mortality from COVID-19 (6, 7), adults with diabetes were more worried about contracting COVID-19 than their peers without chronic disease (8, 9). Increased pandemic worry, in turn, was associated with poorer psychosocial health and feelings of isolation and loneliness (10). Further, nearly half of adults with diabetes reported that the pandemic made diabetes management more difficult (11), and higher A1c was observed in those who reported less physical activity and an unhealthy diet during the pandemic (12). Increases in diabetes-related stress during the pandemic were also linked with higher A1c (11). To date, similar information has not been reported in teens, which represents a critical gap in the literature considering that prior to the pandemic over 80% of U.S. adolescents with T1D were not meeting glycemic targets (13).

The current study was designed to describe the impact of the COVID-19 pandemic on psychosocial health and diabetes management in an adolescent population with T1D and elevated diabetes distress. Specifically, we aimed to explore the effects of the COVID-19 pandemic on teens with T1D, to understand what coping strategies they used to manage stress, and to describe the impact of the pandemic on diabetes management. We anticipated that teens with T1D would report that the COVID-19 pandemic significantly impacted various aspects of their lives and their T1D self-management.

## Materials and Methods

Data for this study were part of a larger set of baseline measures for a psychosocial intervention trial for teens with T1D and elevated diabetes distress that was ongoing during the start of the COVID-19 pandemic (clinicaltrials.gov registration: NCT03847194). Quantitative and qualitative data were collected using REDCap electronic data capture tools hosted at the University of Washington (14, 15). This research was approved by the relevant Institutional Review Boards.

### Participants

Participants were eligible for the trial if they: 1) were aged 13-18 years old, 2) had a duration of T1D ≥ 12 months, 3) reported elevated diabetes distress within the prior 12 months (Problem Area in Diabetes Scale-Teen Version [PAID-T] (16) score ≥30), 4) spoke English fluently, and 5) were cognitively able to participate in intervention sessions and complete written surveys. Participants were screened through the diabetes/endocrinology clinics at their respective institutions, and recruitment primarily occurred *via* phone/video chat, although there were options for recruitment during outpatient or telehealth visits as was desired/appropriate. For participants under 18, written assent from the participant and written consent from the parent/legal guardian was provided. For participants aged 18, written consent was provided. We added a questionnaire related to the COVID-19 pandemic to baseline surveys in August 2020 and administered it to all newly enrolled study participants through June 2021. Participants were provided 6 weeks to complete their baseline survey and A1c. Participants received a monetary incentive ($20) for completion of their baseline survey and were eligible for further incentives for subsequent surveys (up to $80). Participants (n=122) completed the COVID-19 questionnaire as part of baseline measures prior to randomization and intervention.

### Measures

Demographic data were collected *via* a self-report survey that participants completed as part of baseline measures. Health insurance, diabetes duration, A1c, insulin pump use, and CGM use were abstracted from electronic health records.

The Problem Area in Diabetes Scale-Teen Version (PAID-T) was utilized as a screening measure to assess the self-perceived emotional burden of living with diabetes (16, 17). The 14-item scale is the only measure of diabetes distress developed and validated purposely for use with teens. Patients responded on a 6-point Likert scale (1=not a problem, 6=serious problem), and higher scores represent greater distress.

The COVID-19 questionnaire is a 26-item self-report questionnaire developed for this study to assess perceived impact of the COVID-19 pandemic on participants. These items were adapted from other measures of COVID-19 stress and impact that were newly developed at the start of the pandemic, including the COVID-19 Exposure and Family Impact Survey (18) (CEFIS-19) and the COVID-19 Impact Measure (19). Items from these measures most relevant to the study aims were utilized, and minor wording changes, as were appropriate for the target population, were made by content knowledge experts on the study team.

In the first 11 questions, items assess worry/anxiety related to COVID-19, life events as a result of COVID-19 (e.g., missed school), lifestyle changes, and known COVID-19 symptoms/diagnosis for self, family, and important others. Sample items included: “*Overall, how worried or anxious have you been about the COVID-19 pandemic?”* and “*How have changes in your life caused by COVID-19 impacted you?”* Two open-ended question allowed for participants to comment on their general experiences of the COVID-19 pandemic, including: *1) Tell us about other effects of COVID-19 on yourself and/or your family, both negative and/or positive; 2) What is helping you through the COVID-19 pandemic?* The following 15-items focused on participants’ appraisal of how the COVID-19 pandemic had affected their T1D management. 14 of the items were on a 5-point Likert scale (1=not at all; 5=extremely) and pertained to key domains of diabetes management. Items included the same stem (“*Since the COVID-19 pandemic, I…)* and were phrased in both negative *(“…have struggled to properly manage my diabetes.”)* and positive directions *(“…have found it easier to manage my diabetes.”).* Domains included overall management, food/eating, physical activity, diabetes supplies, blood glucose variability, access to healthcare team, and family management. The remaining question was open-ended and asked participants: *3) In what ways has COVID-19 impacted your T1D management?*

### Analysis Plan

Following the Standards for Reporting Qualitative Research (SRQR) (20), the qualitative study team included researchers with training in psychology (MO, MH, VC, JYF), endocrinology (FM, DD), medical anthropology and global health (KR), and health services science with expertise in qualitative research (KB, FM). Three members of the study team (MO, VC, KR) were trained to code the qualitative data under the supervision of the team’s qualitative lead (KB). The lead coder (MO) met with the qualitative lead to discuss a data analysis plan, to share codes, and to get feedback on the process. The lead coder periodically shared results, received feedback about codes, and identified themes with members of the investigator team (JYF, MH, FM, DD). The full writing group provided input into interpretation of codes to assure clinical relevance.

At the time of data analysis, existent literature on the COVID-19 pandemic’s impact on teens with T1D was lacking. As such, we decided to take an inductive qualitative approach and used conventional content analysis (21). Each question was individually analyzed and open coding was conducted to create a codebook for each of the three questions. The lead coder generated initial codebooks from the response data. The three coders (MO, VC, KR) independently applied the codebook categories to all of the responses in separate Microsoft Excel spreadsheets and the coding team met regularly to identify discrepancies among coders and refine the codebook (e.g., by adding new codes for ideas that were not captured by existing codes). Multiple codes could be applied to a single response. Since both Question 1 (Effects of COVID-19) and Question 3 (COVID 19’s Impact on T1D management) generated both positively and negatively worded responses, after primary codes were applied, responses were coded with sub-codes of “positive” or “negative” if the participant added a decipherable valence to their response.

After each iteration, each coder independently recoded the transcripts using the updated codebook. This process continued until there were minimal (<5) discrepancies across all three coders for both codes and sub-codes. Any discrepancies that could not be resolved within the coding team were escalated to the team’s qualitative lead for adjudication. Once final coding occurred, codes across all three questions were tallied to identify the most widely endorsed code categories within each response set. The full research team then considered how the codes related to one another, to theories of resilience, and to team members’ clinical experiences with teens with T1D during the pandemic to identify meaningful themes.

Survey items regarding COVID-19 impacts on diabetes management were collapsed from a 5-point Likert scale to 3 categories (Not at all/Slightly, Moderately, Very/Extremely) based on distributions for ease in interpretation and presentation. Two questions *(“I have struggled to properly manage my diabetes”* and *“I have noticed more fluctuations/variability in my blood glucose levels”*) were dichotomized as Not at all/Slightly versus Moderately/Very/Extremely because the distribution suggested a natural division between people who experienced little to no difficulty versus those reporting greater impact. A1c at enrollment was categorized as <8% * vs.* ≥8% as an indicator for elevated A1c. Participant demographics, A1c and survey responses related to general impacts of COVID-19 were summarized descriptively using frequencies and percentages for categorical variables and means with standard deviations for quantitative variables. Associations between survey responses and A1c levels (<8% * vs.* ≥8%) were assessed using Chi square tests.

## Results

### Participant Demographics

Participants (n=122) were 56% Female, 2% American Indian/Alaska Native, 5% Asian, 11% Black/African-American, 1% Native Hawaiian/Pacific Islander, 80% White, and 7% Other (participants could endorse multiple racial identities). 18% of the sample indicated Hispanic/Latino ethnicity. Participants had a mean A1c of 8.5% (2.1%), 71% used an insulin pump, and 76% used a CGM (**Table 1**).

**Table 1:** Sample characteristics (N=122).

*Demographic Characteristics*	
Age 13-17 years	88%
Age 18 years	12%
Age in years, median (IQR)	15 (14-16)
Gender	
Male	40%
Female	56%
Other	4%
Race*	
American Indian/Alaska Native	2%
Asian	5%
Black/African American	11%
Native Hawaiian/Pacific Islander	1%
White	80%
Other	7%
Ethnicity	
Hispanic/Latino Ethnicity	18%
Public Health Insurance	29%
Site	
A	52%
B	48%
*Diabetes Characteristics*	
A1c, mean ± SD	8.5 ± 2.1
Duration in years, median (IQR)	5.9 (3.4-8.9)
Pump use	71%
CGM use	76%

*Participants could indicate multiple responses.

### General Impact of COVID-19

*Prevalence of COVID-related events.* Regarding incidents related to COVID-19, 11% (13/122) of teens in this sample reported a diagnosis of COVID-19 themselves, and 2% (2/122) were hospitalized. One half (63/122) of teens had a family member or other important person diagnosed, 19% (23/122) had a family member or other important person who was hospitalized, and 12% (15/122) of teens reported that they had a family member or other important person in their life die due to COVID-19.

**Figure 1:**
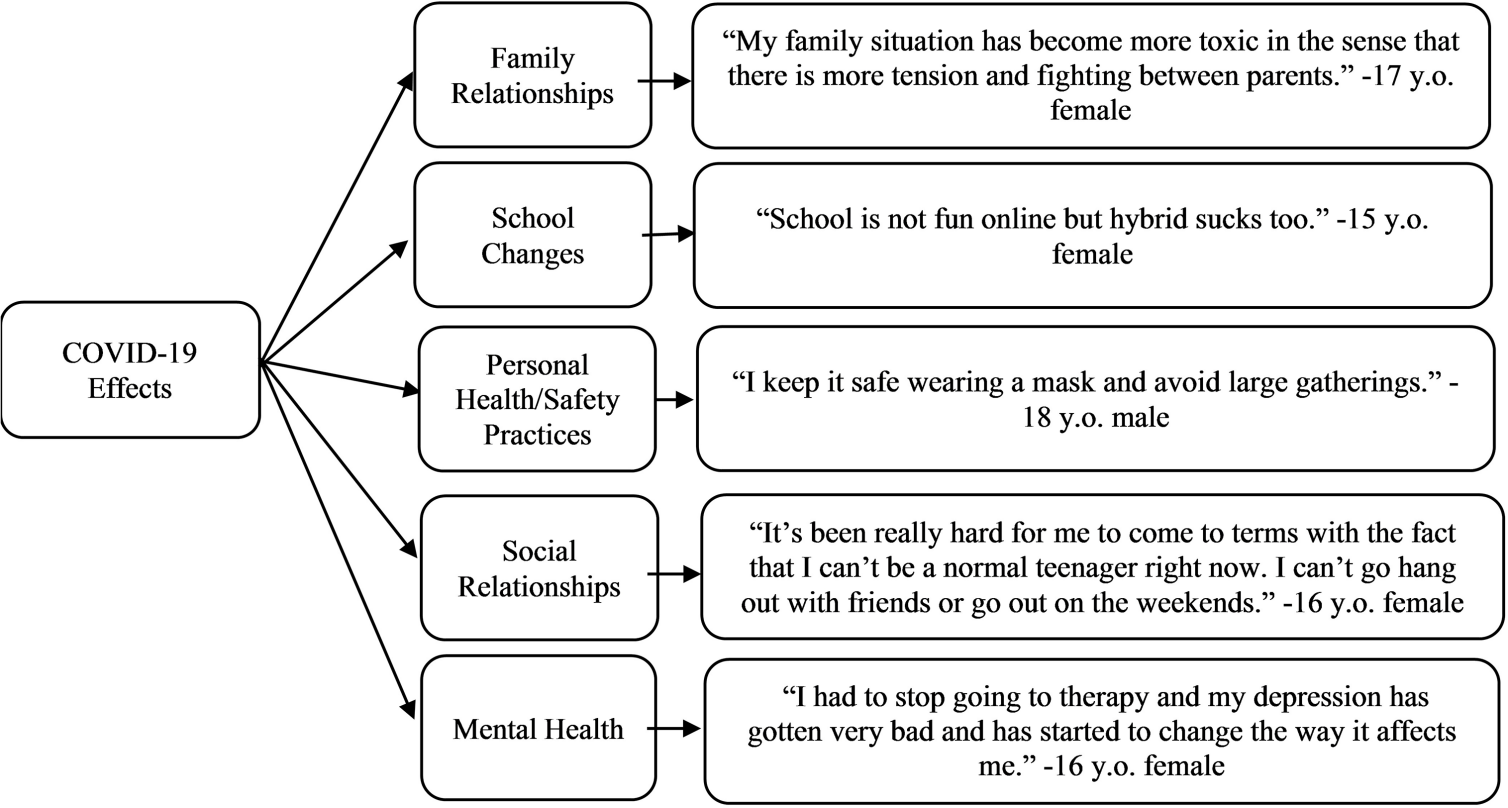
Most frequently observed code categories of teen-reported COVID-19 effects with example quotes.

*Personal impacts and responses to COVID-19.* On the quantitative survey, 44% (54/122) of teens reported that they were not at all or slightly worried/anxious about the COVID-19 pandemic, 37% (45/122) reported that they were moderately worried/anxious and 19% (23/122) reported that they were very or extremely worried/anxious. In response to the open-text question regarding negative or positive effects of COVID-19 on the teen or their family (Q1), there were 14 code categories (**Table 2**). The most frequently observed code categories were: Family Relationships (n=37), School Changes (n=21), Personal Health and Safety Practices (n=21), Social Relationships (n=18), and Mental Health (n=17) (**Figure 1**). Changes in family relationships was a widely endorsed effect of COVID-19, although some teens found both positive and negative effects. For example, one 13-year-old gender non-binary teen noted, “Staying at home together all the time has caused tension between my family, but we have also grown closer.” Several teens noted the changes to their schooling, which were described as overwhelmingly negative. A 14-year-old male teen simply described, “School in my opinion is worse now (with it being online)…” Participants also discussed negative effects to their social relationships and mental health. One 13-year-old female teen described both noting, “There are not any positive effects. I can’t see my friends and my dog got an … injury that we can’t get treated because of COVID. There is nothing to look forward to. Every day is the same … my mental health has worsened…”

**Table 2:** Code categories, code definitions, and number of times coded.

Codes	Code Definitions	Number of Times Coded
*Question 1 (Q1): Effects of COVID-19 (General)*		
Family	Included references to family relationships, both implied or explicit	37
School	Included references to school, school-related activities and/or extracurricular activities associated with school	21
Personal Health and Safety Practices	Included references to health and safety practices and not going out/leaving home	21
Mental Health	Included references to stress and/or any mental health symptoms	17
No Effect	Included language that suggests that COVID-19 has not made an impact on their lives	12
Cancellation	Included specific references to cancelled events (not including travel)	10
Physical	Included references to any aspect of physical health or fitness which cannot otherwise be accounted for as diabetes care	8
Perspective/Cognitive Shifts	Included references to changes in thinking and/or their outlook on the world	7
Financial	Included references to money/finances or implied financial changes	7
Comfort/Flexibility	Included references to flexibility, absence of routine stressors, and/or comfort related places	5
Maturation	Included references to growth and/or development	3
Diabetes Care	Included references to any aspect of diabetes management	3
Loss	Included references to mortality due to COVID-19 or other factors	2
Travel	Included specific references to travel	1
*Question 2 (Q2): What’s Helping Them Through*		
Relationships	Included noted relationships, correspondence or connection with friends, family, pets, teachers, doctors, etc.	65
Stress Management *via* Entertainment, Hobbies, & Exercise	Included references to leisure activities (e.g., social media, video games, art, and exercise in any form)	43
School	Included references to school, school-related activities (e.g., studying) and/or extracurricular activities associated with school (e.g., band practice)	11
Looking Forward/Perspective	Included references to positively anticipating the future and/or thinking about the pandemic in a way that is beneficial to the individual	9
Personal Health and Safety Practices	Included references to health and safety practices and not going out/leaving home	8
Nothing	Included references to no coping resources/nothing is helping them get through	6
Comfort/Flexibility	Included references to flexibility, absence of routine stressors, and/or comfort related places	5
Distraction	Included references to not thinking about the pandemic or participating in activities to avoid thinking about the pandemic	5
Personal Development/Maturation	Included references to bettering oneself, growth, and/or accumulating knowledge	4
Work	Included references to work or work-related activities	3
Religion/Spirituality	Included references to religiosity, spirituality or god	2
Introversion/Appreciating being Alone	Included references to introversion and/or enjoying alone time	2
*Question 3 (Q3): Effects of COVID-19 (T1D Management)*		
No Effect	Included language that suggested that there has been no impact of T1D management as a function of COVID-19	50
Blood Glucose Management	Included references to managing blood sugar	18
Food	Included references to food and/or eating	14
Self-Care	Included references to taking care of the self (without any specific references to any one aspect of diabetes management)	13
Diabetes Appointments	Included references to diabetes appointments and diabetes care team (including telehealth)	13
Personal Health and Safety Practices	Included references to health and safety practices and not going out/leaving home	13
Exercise	Included references to exercise and/or physical activity	12
Mental Health/Stress	Included references to stress or any mental health symptoms	7
Motivation	Included references to their desire or willingness to manage their diabetes	5
Unknown	Included language that suggests that patient is unclear or does not know about the impact of COVID-19 on their diabetes management	3

In response to the question of “What is helping you through the COVID-19 pandemic?” (Q2), there were 12 code categories (**Table 2**). Most commonly, teens reported that Relationships (n=65) and Stress Management *via* Entertainment, Hobbies, and Exercise (n=43) were helping them through. For example, a 14-year-old female teen described that “being able to still talk to people I love through facetime and text” was helping them get through, while another 16-year-old female teen described multiple behavioral strategies, “Increased free time to do more exercise and hobbies. Running and hiking have been good stress-relievers. I have also had more time for baking and reading.” Six participants noted that there was nothing that was helping them through the COVID-19 pandemic.

**Figure 2:**
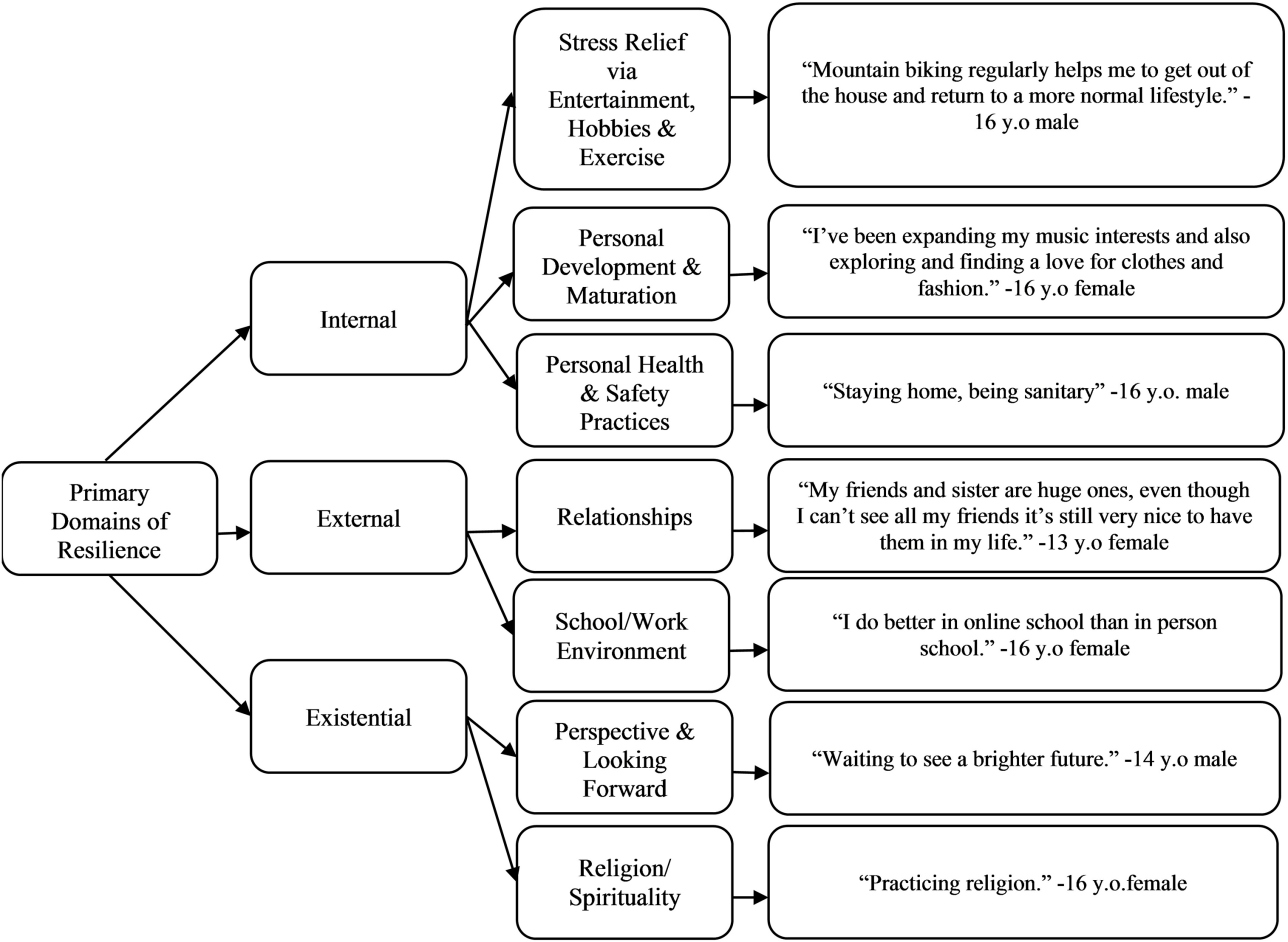
Themes and code categories of teen-reported resilience with example quotes.

From the codes, we identified overarching resilience themes consistent with stress and coping (22) and resilience theories (23, 24). These themes were: 1) Internal Resilience Resources, which referred to personal learned skills and behaviors (i.e., *what* helped the teen navigate COVID-19), 2) External Resilience Resources, which referred to social support and community resources (i.e., *who* helped the teens navigate COVID-19) and 3) Existential Resilience Resources, which referred to meaning-making, faith, religious, and spiritual resources (i.e., finding a *why* in navigating COVID-19) (**Figure 2**).

### Impact of COVID-19 on T1D Management

**Figure 3:**
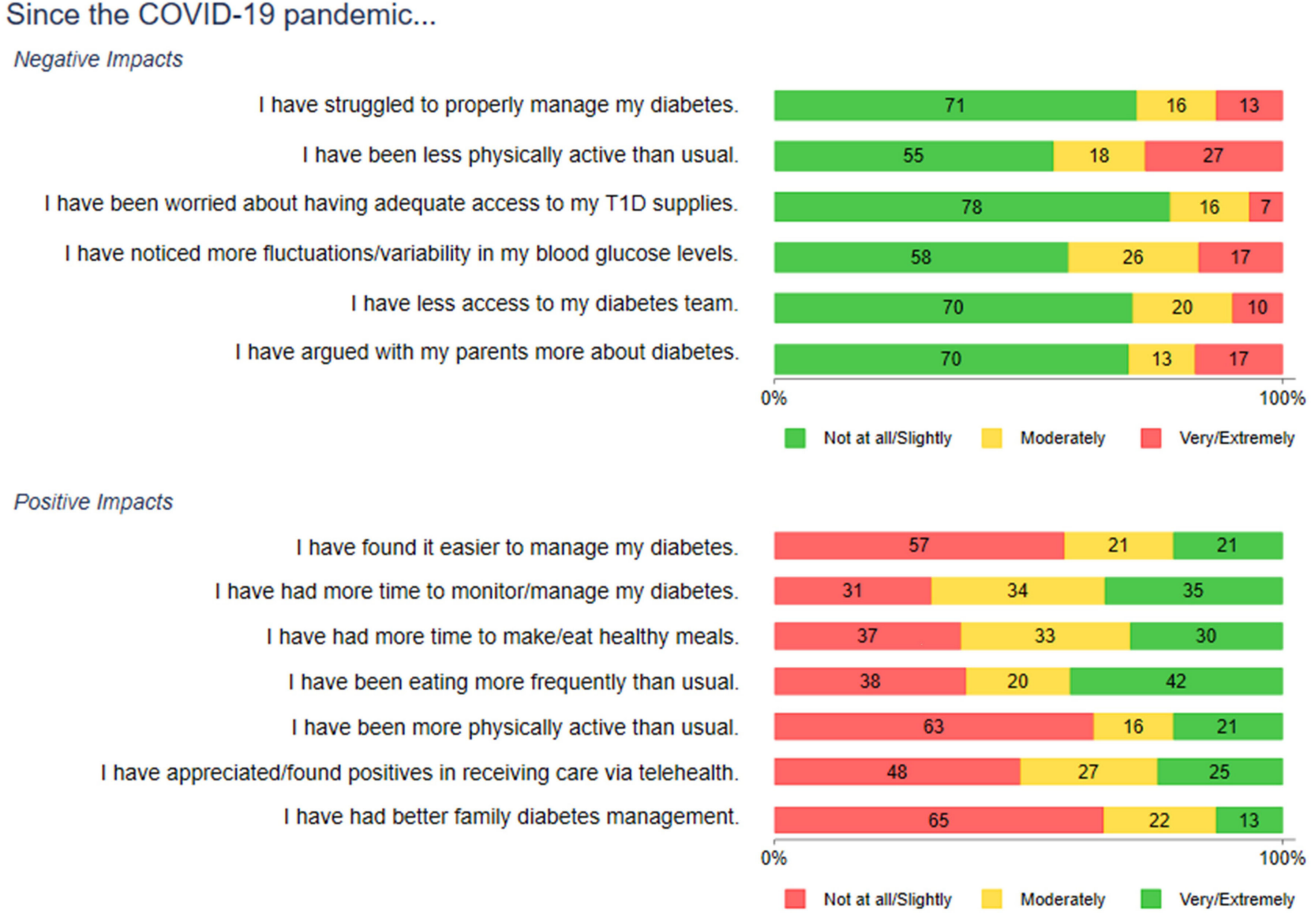
Negative and positive impacts of COVID-19 on diabetes management. Numbers shown are percentages.

**Figure 4:**
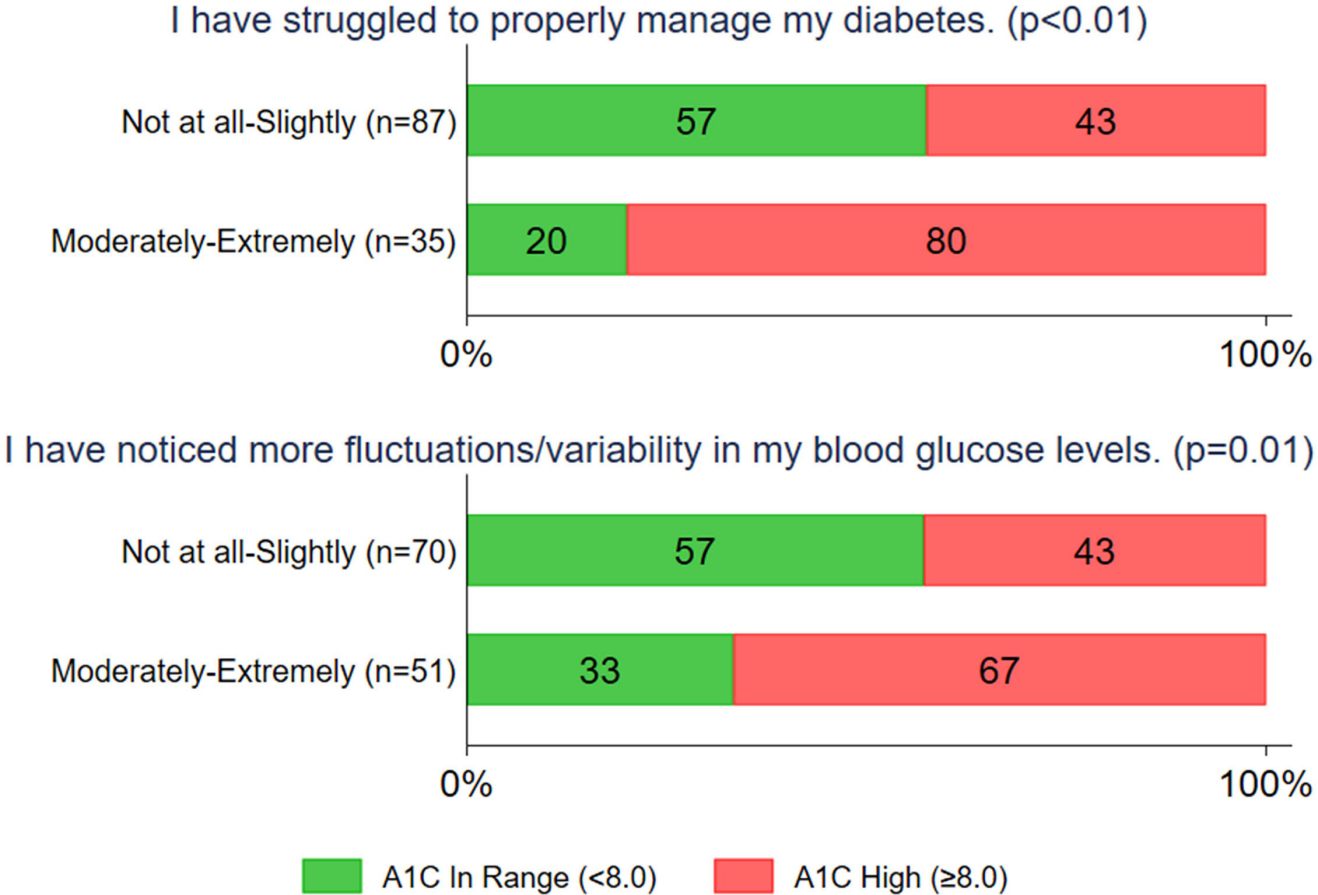
Percentages with <8% and ≥8% A1C among subgroups of respondents who did or did not report problems with diabetes management since the COVID-19 pandemic. P values are from Chi square tests.

On the quantitative survey, the majority of teens (71%) reported that they were not at all or only slightly affected by COVID-19 in terms of properly managing their diabetes. Most teens endorsed that they had continued access to their diabetes care team (70%) and that they were not arguing with their parents more about diabetes during the COVID-19 pandemic (70%) (**Figure 3** and **Supplemental Table 1**). Of the 29% of teens who experienced increased (moderate to extreme) difficulty were more likely to have A1c ≥ 8% (80% versus 43%, p<.01). The 42% of teens who reported greater fluctuations in blood glucose levels also were more likely to have A1c ≥ 8% (67% * vs.* 43%, p=.01) (**Figure 4**).

**Figure 5:**
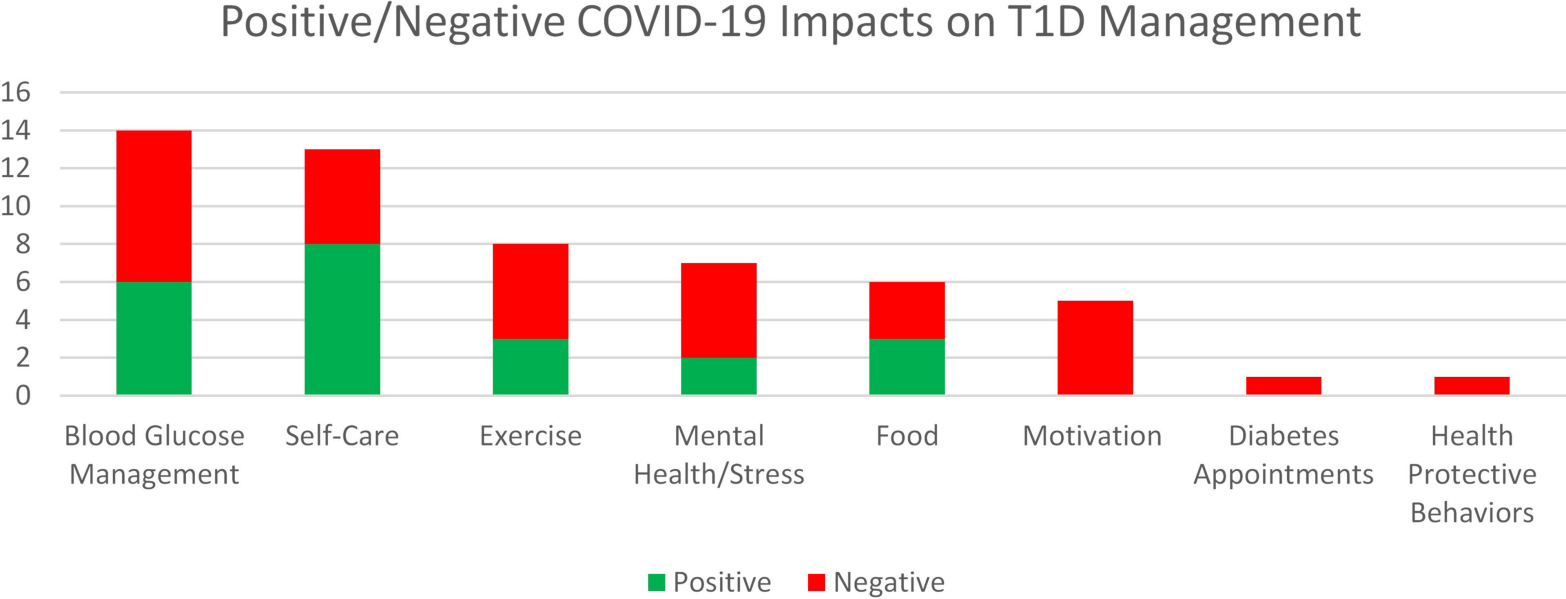
Positive and negative teen comments about COVID-19's impact on Type 1 Diabetes management. Numbers shown are counts.

In response to the open-text question of, “In what ways has COVID impacted your T1D management?,” there were 10 code categories (**Table 2**). Most commonly, when asked in an open-ended fashion, teens reported that there was no effect of COVID-19 on their diabetes management (n=50). The next most widely endorsed domain was blood glucose management (n=18). For example, one 16-year-old male teen noted, “I’ve had more time to focus on my blood sugars,” while another 16-year-old gender non-binary teen described, “It’s [COVID-19] also been hard emotionally and diabetes management is much harder when it’s difficult to find the energy to care about blood sugars.” If participants indicated a positive or negative valence to their response, results were plotted in **Figure 5**. Overall, for those whose T1D management was affected by the COVID-19 pandemic, participants described more negative impacts than positive impacts.

## Discussion

The purpose of this study was to explore the effects of the COVID-19 pandemic, to understand what coping strategies were utilized, and to describe the impact of the pandemic on diabetes management for a diverse sample of teens with T1D and elevated diabetes distress participating in a clinical trial. There were pervasive impacts of the COVID-19 pandemic in our sample—the majority of teens reported moderate to high anxiety about the pandemic and had direct knowledge of an important person in their lives having COVID-19. More than 1 in 10 teens in our study were diagnosed themselves or had a family member or other important person die due to COVID-19. Teens also highlighted stressors in a wide range of areas due to the COVID-19 pandemic, including most commonly with family, engaging in personal health and safety practices (e.g., social distancing), in their social relationships, school, and in their own mental health—all of which are life domains that contain social and relational elements. This finding is in line with other emergent research that suggests that teens have experienced more family conflict and difficulty navigating peer relationships during the COVID-19 pandemic (25, 26). COVID-19 and associated safety measures (e.g., quarantine) have had a crushing social impact globally (27), which may be especially concerning for adolescents who are at a key time of social and emotional development (28). This may be a driving cause as to why teens are at risk of developing mental health symptoms during the COVID-19 pandemic (29).

Despite the widespread impact of the COVID-19 pandemic on many parts of life, most teens generated specific strategies, skills, and resources that were helping them to navigate the COVID-19 pandemic. Thematically, many of these resilience strategies were consistent with existent theories of stress, coping, and resilience literature, which suggest that teens, especially in the context of chronic disease, will accumulate and apply resources to navigate challenges as they arise (22–24). Some theories of resilience further suggest categories of resilience resources, which fall into individual, community, and existential domains (23, 30). Teens reported strategies across all these domains, demonstrating pervasive use of internal, external, and existential resilience resources; this provides support that employing resilience resources is both attainable and a “universal” response to stress (23). This pattern is particularly notable and supportive of resilience, given that these were teens with elevated diabetes distress. Teens in our sample predominantly reported engaging in personal behavioral strategies, such as using technology or pursuing hobbies, and/or relying on existing social support structures. This finding provides continued rationale for stress management and resilience interventions that bolster personal and existential resources for high-risk groups. These intrapersonal skills can be utilized in multiple settings and life domains, which aligns with teens’ reports that they felt stress in multiple arenas of their lives throughout the COVID-19 pandemic.

Contrary to the literature describing adults with diabetes (11), for many teens with T1D, diabetes management was *not* one of the major sources of stress and was not significantly impacted by the pandemic. This suggests that teens’ perceived diabetes resilience was high during the pandemic, even higher than what has been reported in adult populations with diabetes (11). Although conclusions cannot be drawn from this study about why this is the case, it is plausible that aspects of the COVID-19 pandemic response in the United States may have facilitated resilience in diabetes management for some. For example, teens may have had more hands-on parental involvement and support for daily diabetes management tasks while at home and may have spent less time in environments that introduce barriers to consistent diabetes self-management (e.g., school, sports, social gatherings, etc.). Together, changes in daily routines may have reduced vulnerability to blood glucose variability and made it easier for teens and families to manage diabetes. This finding is consistent with positive psychology and diabetes literature that suggests that people with diabetes draw on strengths and exhibit resilience during times of stress (24, 31).

Although not the predominant experience of the teens in this study, it was notable that over a quarter of participants reported serious impacts of the COVID-19 pandemic on diabetes management. Further, many of these participants were already struggling with diabetes management given our finding that adolescents in this subgroup were more likely to have higher A1c levels. This aligns with data demonstrating associations between stress, diabetes distress, and A1c generally (2) and extends the results to stress specifically related to the COVID-19 pandemic (11). Our results suggest that teens may be more likely to feel the effects in the following areas: blood glucose management, self-care, exercise, and mental health. With this knowledge, diabetes teams can identify target areas for intervention and collaborate with patients and families to find workable solutions in light of the specific stressors the teen may be facing.

### Limitations

The nature of this study is descriptive and limits the extent to which we may be able to make any causal inference about this population. Additionally, patients completed the COVID-19 survey as early as August 2020 and as late as June 2021. Major shifts in the COVID-19 pandemic had occurred prior to the start of the study period and occurred during the study period, such as the availability of the COVID-19 vaccine, which may have differentially impacted participant responses on the COVID-19 questionnaire. In addition, throughout our data collection period, there were several notable co-occurring stressors, such as racial tensions in summer 2020, political unrest related to the 2020 election, and multiple climate disasters in our respective regions, any of which could have impacted stress and coping but were not assessed in this study.

Further, due to the free response nature of the qualitative questions, responses were often brief, and we were unable to seek clarification about their responses or follow up with probing questions. This limited our ability to deeply explore the impact of COVID-19 and contributes only a basic understanding of what teens with T1D were experiencing. It is also possible that some patients felt uncomfortable sharing about sensitive topics in this format, which may have restricted the range of responses.

This study was conducted at two large academic pediatric diabetes centers in urban centers of two different areas of the United States (Pacific Northwest and Gulf Coast). While this allowed for a culturally, racially, ethnically, and socio-economically diverse sample, the results may not be generalizable to adolescents who live in other areas or whose care is delivered in other settings.

### Future Research and Clinical Directions

To more fully understand the phenomena observed here, future studies should include qualitative interviews about stress and resilience both generally and related to specific adverse circumstances, such as future public health crises. Interviews may provide more detailed data about which types of stressors tend to derail T1D management and how teens cope with those stressors.

This study may help to inform stress management and resilience interventions for teens with T1D. Such interventions may benefit from building on teens’ existing coping skills (e.g., behavioral and social support strategies) and introducing intrapersonal and existential/meaning-making skills, which were less common in our sample. Given the social nature of many of the teens’ stressors, they may benefit from additional support and resources when re-integrating into contexts that were paused during the COVID-19 pandemic. From a strengths-based perspective, it may be valuable to help teens recognize the ways they *already* successfully manage stress, both in general and specific to their diabetes management, and to promote recognition of which resilience resources benefit them most and when.

Finally, results from this study highlight the possible care needs for teens with T1D who are both stressed about their diabetes and experience difficulty managing their disease. Future studies may systematically explore this sub-group’s experience of the COVID-19 pandemic. Clinical diabetes teams may consider specifically including questions about the COVID-19 pandemic or other life stressors in clinic surveys, including if and how the COVID-19 pandemic has affected their care routines. Teens in this group may benefit from increased access to services and tailored health interventions to address stress and diabetes management. Existent strengths-based intervention for teens with diabetes (32, 33), which both explore strengths and identify areas for growth, may be particularly beneficial for this higher risk group.

## Conclusions

The COVID-19 pandemic has undoubtedly made an impact on teens with T1D, and our quantitative and qualitative findings reveal that teens with T1D felt the effects of COVID-19 predominantly in social aspects of their lives. Despite significant changes to major domains of their lives, many teens reported that their T1D management was protected and they described using coping strategies that were helping them through this stressful time, demonstrating diabetes resilience. However, for those whose T1D management was negatively impacted by COVID-19, higher A1c was more common, suggesting a need for focused follow-up by diabetes care teams.

## Data Availability Statement

The raw data supporting the conclusions of this article will be made available by the authors, without undue reservation.

## Ethics Statement

The studies involving human participants were reviewed and approved by Seattle Children’s Institutional Review Board (IRB of Record); Baylor College of Medicine Institutional Review Board (Relying Site). Written informed consent to participate in this study was provided by the participants’ legal guardian/next of kin or self (if participant had reached the age of majority).

## Author Contributions

All authors contributed to either data collection and/or interpretation. JY-F is principal investigator of this study and oversaw all aspects of the research. MO’D was involved in data collection, analysis, and crafted an initial draft of this paper. MH, FM, DD, and AR are co-investigators of the study and have contributed to study design, oversight, and editing. MB and CZ are biostatisticians and contributed to quantitative analysis. KB is a qualitative expert and contributed to oversight of qualitative analyses. All authors have reviewed this manuscript and approved the submitted version.

## Funding

This study was funded by the National Institutes of Health, National Institute of Diabetes and Digestive and Kidney Diseases (NIDDK). Project Number: R01DK121224.

## Conflict of Interest

DD serves as an independent consultant for Dexcom and Insulet outside the submitted work.

The remaining authors declare that the research was conducted in the absence of any commercial or financial relationships that could be construed as a potential conflict of interest.

## Publisher’s Note

All claims expressed in this article are solely those of the authors and do not necessarily represent those of their affiliated organizations, or those of the publisher, the editors and the reviewers. Any product that may be evaluated in this article, or claim that may be made by its manufacturer, is not guaranteed or endorsed by the publisher.
