# Supplementary material for: “It Just Kind of Feels Like a Different World Now:” Stress and Resilience for Adolescents With Type 1 Diabetes in the Era of COVID-19
Source: Front Clin Diabetes Healthc. 2022 Feb 21;3:835739. doi: 10.3389/fcdhc.2022.835739 (PMC10012077; doi:10.3389/fcdhc.2022.835739)
Supplement: Supplementary file 1 [file Table_1.docx]

Supplemental Table 1. Impact of COVID-19 on key aspects of T1D management.

| Since the COVID-19 pandemic… | Not at all/Slightly | | Moderately | | Very/Extremely | |
| --- | --- | --- | --- | --- | --- | --- |
| I have struggled to properly manage my diabetes. | **71%** | (87/122) | **16%** | (19/122) | **13%** | (16/122) |
| I have found it easier to manage my diabetes. | **57%** | (69/121) | **21%** | (26/121) | **21%** | (26/121) |
| I have had more time to monitor/manage my diabetes. | **31%** | (37/120) | **34%** | (41/120) | **35%** | (42/120) |
| I have had more time to make/eat healthy meals. | **37%** | (44/120) | **33%** | (40/120) | **30%** | (36/120) |
| I have been eating more frequently than usual. | **38%** | (46/122) | **20%** | (25/122) | **42%** | (51/122) |
| I have been less physically active than usual. | **55%** | (67/122) | **18%** | (22/122) | **27%** | (33/122) |
| I have been more physically active than usual. | **63%** | (76/121) | **16%** | (19/121) | **21%** | (26/121) |
| I have been worried about having adequate access to my T1D supplies. | **78%** | (95/122) | **16%** | (19/122) | **7%** | (8/122) |
| I have noticed more fluctuations/variability in my blood glucose levels. | **58%** | (70/121) | **26%** | (31/121) | **17%** | (20/121) |
| I have less access to my diabetes team. | **70%** | (86/122) | **20%** | (24/122) | **10%** | (12/122) |
| I have appreciated/found positives in receiving care via telehealth. | **48%** | (59/122) | **27%** | (33/122) | **25%** | (30/122) |
| I have argued with my parents more about diabetes. | **70%** | (85/122) | **13%** | (16/122) | **17%** | (21/122) |
| I have had better family diabetes management. | **65%** | (79/122) | **22%** | (27/122) | **13%** | (16/122) |
